# Guidelines update

**DOI:** 10.1186/1471-227X-15-S1-A15

**Published:** 2015-06-24

**Authors:** Oktay Demirkiran

**Affiliations:** 1 Department of Anaesthesiology and Intensive Care, Istanbul University Cerrahpasa Medical School Hospital, Istanbul, Turkey

## 

In cardiac arrest patients the primary goal is to restart the heart, return the patient to life, and keep the brain intact. In 1960 a landmark article described the outcome in CPR [1]. In 1964 Peter Safar published the first integrated approach to cardiac arrest, and recommended therapeutic hypothermia (TH) for support recovery [2]. These two studies merged in the first American Heart Association guidelines for the treatment of cardiac arrest patients [3]. The most recent update of the ERC and American Heart Association guidelines were published in November 2010. The use of TH in cardiac arrest patients developed after two cornerstone studies which showed good neurological outcome when the body temperature decreased to 32 to 34°C after out-of-hospital cardiac arrest [4,5]. Hypothermia can prevent or reduce cellular damage in the post-cardiac arrest period [6]. Current resuscitation guidelines recommend use of TH as soon as possible following return of spontaneous circulation [7]. Most TTM protocols call for induction with cold intravenous saline and surface cooling with cold packs while TH devices are being applied. Since then TTM of 32 to 34°C for 12 to 24 hours has been recommended as part of post-resuscitation care by international guidelines. Frydland and colleagues assessed mild hypothermia in 12 studies in patients with out-of-hospital cardiac arrest and nonshockable rhythms as an initial one [8]. TTM has been recommended for nonshockable rhythms [9]. Some observational studies supported the use of TTM in out-of-hospital cardiac arrest and initial nonshockable rhythms [10,11].

The new resuscitation guidelines will represent the most recent and comprehensive analysis of intubation or supraglottic airway devices, mechanical devices, adrenaline use, telephone CPR, hypothermia/TTM, early PCI, and post-arrest care. In the new guidelines in 2015 there may be answers for the optimal temperature target, duration of TH, and rates of cooling and rewarming for post arrest.

## Financial disclosure

OD has received speaker’s reimbursement from C. R. BARD.
